# A new electro-optical approach for conductance measurement: an assay for the study of drugs acting on ligand-gated ion channels

**DOI:** 10.1038/srep44843

**Published:** 2017-03-21

**Authors:** A. Menegon, S. Pitassi, N. Mazzocchi, L. Redaelli, R. Rizzetto, J. F. Rolland, C. Poli, M. Imberti, A. Lanati, F. Grohovaz

**Affiliations:** 1 San Raffaele Scientific Institute, via Olgettina 60, 20132, Milan, Italy; 2 San Raffaele University, via Olgettina 58, 20132, Milan, Italy; 3 Optotec, Via Zenale 44, 20024, Garbagnate Milanese, Milan, Italy; 4 Axxam SpA, via Meucci 3, 20091, Bresso, Milan, Italy; 5 Valore Qualità, Via Vidari 5, 27100, Pavia, Italy; 6 Assing, Pavia, Viale Indipendenza 11, I-27100, Pavia, Italy; 7 OPEN Sistemi, via Bonomelli 24, 26100, Cremona, Italy

## Abstract

Ligand gated ion channels are involved in many pathophysiological processes and represent a relevant, although challenging, target for drug discovery. We propose an innovative electro-optical approach to their analysis able to derive membrane conductance values from the local membrane potential changes imposed by test current pulses and measured by fast voltage-sensitive fluorescent dyes. We exploited the potential of this proprietary method by developing a drug testing system called “ionChannel Optical High-content Microscope” (*ionChannelΩ*). This automated platform was validated by testing the responses of reference drugs on cells expressing different ligand-gated ion channels. Furthermore, a double-blind comparison with FLIPR and automated patch-clamp was performed on molecules designed to act as antagonists of the P2RX7 receptor. *ionChannelΩ* proved highly reliable in all tests, resulting faster and more cost-effective than electrophysiological techniques. Overall, *ionChannelΩ* is amenable to the study of ligand gated ion channels that are receiving less attention due to limitations in current assays.

A fine control of membrane ion fluxes through the ion channels is a basic requirement for the maintenance of cellular homeostasis and it is also at the basis of specialized cellular functions as important as neurotransmitters/hormones release, excitability control, gene activation etc. Accordingly, the notion that alterations in the control of ionic fluxes can be associated with pathogenic mechanisms makes ion channels important therapeutic targets for the treatment of a large number of different pathologies^1^,^2^.

Despite the scientific, social, and even commercial relevance of drugs able to modulate ion channel activity, the process of their discovery suffers from intrinsic problems. Several assays have been proposed to evaluate ion channel activity, but a gold standard is not yet available in drug screening for all ion channel targets. Ion-luminescence or ion-fluorescence assays are widely used for high throughput screening (HTS) but mainly limited to the analysis of calcium-permeable ion channels^3^. The use of non-physiological surrogate ions is also exploited in drug screening since it allows higher signal-to-noise ratio. Fluorescent thallium flux assays are used to study both potassium- and sodium- selective channels, although they are not immune from problems, such as influence of off-target pathways and high toxicity of thallium^3^. Similarly, quenching of mutated yellow fluorescent protein (YFP) by iodide flux is employed for chloride channel screening^3^.

To obtain better qualitative and quantitative information on molecules acting on ion channels, industry has recently turned the attention back to electrophysiology. Manual patch-clamp can measure small ion currents with sub-millisecond temporal resolution, but suffers from being far too slow, laborious and expensive. Higher time efficiency has recently been achieved by automated patch-clamp instrumentation, even though the HTS requirements for drug screening are not fully satisfied, particularly for ligand-gated channels^4^.

Optical technologies based on the measure of membrane potential by Voltage-Sensitive Dyes (VSD) have attracted interest for the study of ion channels^3^,^5^,^6^,^7^,^8^. Slow-responding VSDs, although widely used for their high sensitivity, have temporal responses incompatible with the kinetics of most ion channels. An interesting approach that uses electric field stimulation to open voltage-dependent channels, and based on a FRET-VSD to measure membrane potential changes, was proposed, opening the possibility of performing optical electrophysiology for screening purposes^9^. However this innovative approach is not without limitations: membrane potential is not linearly correlated with the ion channel currents^7^ and changes in membrane potentials depend on the membrane orientation within the electric field. In the present study, we report of a new electro-optical screening system, the *ionChannelΩ*, based on a proprietary method^10^ by which it is possible to study a ligand-gated channel, independently of its specific permeability and of the extent of the ionic flux sustained by the electrochemical potential at rest. Finally, we compare data generated by *ionChannelΩ* on different pharmacological targets with those obtained by conventional FLIPR fluorescent assays and automated patch clamp.

## Results

### Effect of electrical field stimulation on local cell membrane potential

The passive properties of the membrane state that upon administration of an extracellular electric pulse, cell membrane becomes hyperpolarized on the side facing the anode and depolarized on the side facing the cathode (Supplementary Figure 1). These local membrane potential changes can be followed optically by the use of a fast-responding VSD (Supplementary Figure 1).

**Figure 1:**
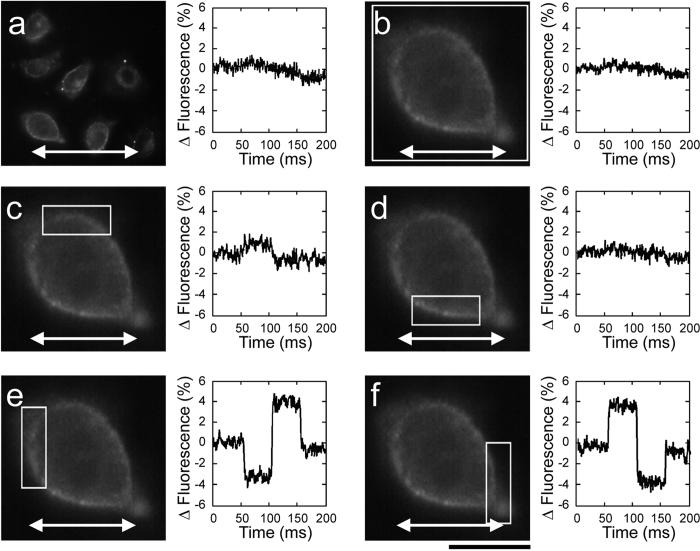
Optical detection of local membrane potential changes imposed by a current pulse. VSD fluorescence intensity changes (% of pre-pulse intensity) were recorded in cellular or subcellular regions (highlighted by squares in the images) of HeLa cells exposed to a biphasic current pulse (100 ms total duration). The average signal recorded from a field containing multiple cells (**a**) or even from a single whole cell (**b**) shows no variations upon stimulation. Small or no variations in membrane potential are observed also on the axis perpendicular to the field direction (**c**,**d**). The strongest changes are observed along the field direction with one end of the cell depolarized and the other end hyperpolarized (**e**,**f**). The line with two arrows indicates field line orientation. Scale bar: 20 μm (**a**); 5 μm (**b–f**).

We investigated membrane potential variations in HeLa cells loaded with di-4-ANEPPS and exposed to symmetric biphasic electric pulses (see Methods). When the mean fluorescence intensity was monitored in a region containing multiple cells (Fig. 1a) or even a single whole cell (Fig. 1b), no changes were observed, because the sum of negative and positive variations cancels out. As expected from the theory, the biphasic electric pulse elicited no effects in the direction perpendicular to the electric field lines (Fig. 1c and d), while caused maximal fluorescence changes in the parallel orientations: a depolarization followed by hyperpolarization at the one side (Fig. 1e), and the opposite at the contralateral side (Fig. 1f).

### Measurement of membrane resistance changes induced by drugs able to alter membrane permeability

Resistance (R) determines the magnitude of the passive changes observed in membrane potential in response to a constant current: the lower the resistance, the lower the membrane potential variation (Supplementary Figure 1). Accordingly, we investigated the possibility of using local membrane potential variations imposed by a test square current pulse to measure changes in membrane resistance. The variation of VSD fluorescence values during the current pulse, were quantified as ΔF, i.e. the difference between the plateau value reached during the electric stimulus (*F*_*S*_) and the pre-stimulus, resting, value (*F*_*R*_) (Supplementary Figure 1). ΔF values were further normalized to their respective resting values (*ΔF/F*_*R*_) to take into account even minimal variations of VSD fluorescence due to dye bleaching.

**Figure 2:**
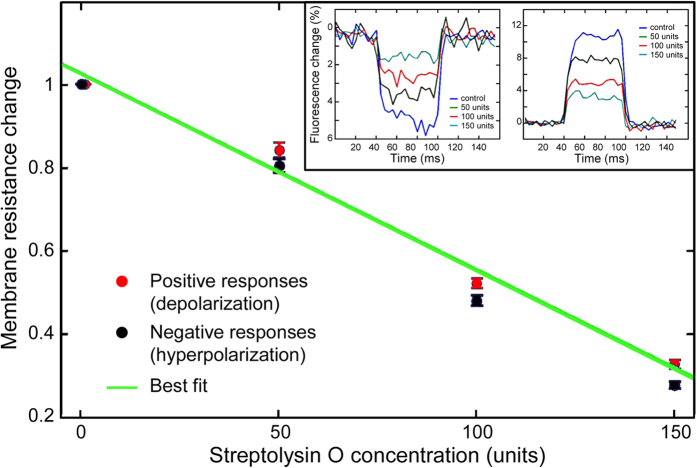
Optical detection of changes in plasma membrane resistance in HeLa cells exposed to streptolysin-O. A culture glass was challenged (by a series of 3 stimuli, with 100 ms interval in between each pulse, to allow cell recovery) before and after administration of increasing concentrations of streptolysin-O. The inset shows examples of the VSD variations imposed by a square pulse in the absence or the presence of different streptolysin-O concentrations. The superimposed traces in the inset show fluorescence changes in two different subcellular regions selected for their high responsivity to the current pulse; on the left and right panels, negative (depolarizations) and positive responses (hyperpolarization) are shown, respectively. With increasing concentrations of the drug, a clear reduction of the plateau levels is observed in both depolarising (left) and hyperpolarising (right) responses. Changes in resistance (normalised to control value) after administration of increasing concentrations of streptolysin-O are shown for both positive and negative variations (red and blue dots respectively). Green line shows the best linear fitting; bars represent the standard errors of each series of 3 stimuli.

The responsivity of HeLa cell membrane to electric square pulses, elicited by a constant current generator, was tested under a conventional microscope before and after exposure to increasing concentrations of Streptolysin-O, a bacterial protein able to insert into the eukaryotic plasma membrane and to form non-selective ion pores that alter the resistance of the membrane. Figure 2 shows how responses were progressively reduced in amplitude when increasing concentrations of Streptolysin-O were administered.

By considering that local responses were elicited by the same pulse of current (I) in control conditions (*ΔV*_*c*_ ≈ *ΔF*_*c*_/*F*_*Rc*_) and after exposure to the drug (*ΔV*_*d*_ ≈ *ΔF*_*d*_/*F*_*Rd*_), and taking into account that a constant current was applied, the Ohm’s law can be applied:


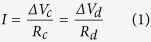


and the following simple relation can be derived:


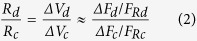


Finally, considering that the conductance (G) is the reciprocal of the resistance, we can also write:


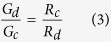


It can be demonstrated that a variation of the resting membrane potential (induced by drug administration) between two successive electric pulses has no influence on the calculation of the resistance/conductance change, provided one operates in the linear region of the spectrum^10^.

Therefore, by measuring local variations of the VSD fluorescence signals, a direct measure of the membrane resistance/conductance changes induced in the same cells upon administration of increasing concentrations of streptolysin-O was obtained. A linear relationship between concentrations and drop in resistance/conductance superimposable for both depolarization and hyperpolarization responses to the current pulse – was observed (Fig. 2).

### *IonChannelΩ*, an automated platform for ion channel drug screening

**Figure 3:**
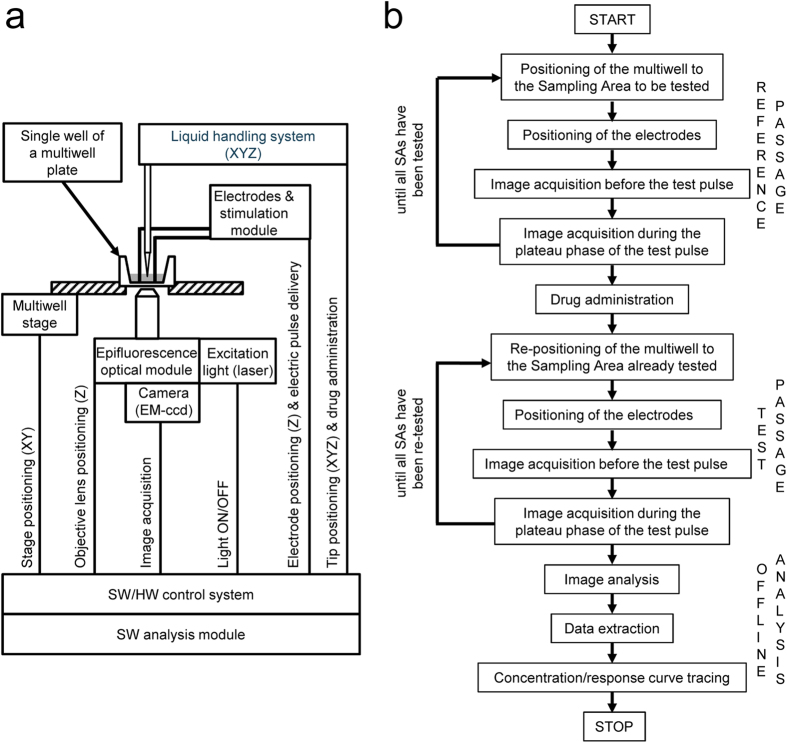
The ionChannelΩ platform for drug screening. Panel a illustrates the Block Diagram of ionChannelΩ. Single- and multi-axis linear stages provide flexible and accurate positioning of the multiwell plates and of the electrodes; a carthesian liquid handler provides proper dispensing of the drug. Sequential and parallel tasks are governed by a control software. The ionChannelΩ controls, in full automation, the following main functions: positioning of the multiwell (96 wells), auto focus, laser excitation, image acquisition, electrode positioning and electric pulse delivery, drug administration. Image analysis, data extraction and calculation of CRCs are performed off line. A graphic interface allows the flexibility necessary to adapt the procedure to the study of specific channels but also guarantees the reproducibility and reliability to perform a fast and robust processing of samples. The functional development of the prototype into an industrialized platform was performed by adopting industry standard quality methodologies. In particular, a Failure Mode and Effects Analysis (FMEA) was applied in various phases of the design and assembly of the instrument to lower the probability of malfunctions and reduce risks. The definition and design of the validation tests were conducted according to the best practice principles (e.g. V-cycle process) and by considering and respecting guidelines, standards and regulations. The typical protocol for ion channel drug screening requires two passages performed in sequence (**b**), each one involving the acquisition of two images (one before and one during the plateau phase of the electric pulse) in up to 9 sampling areas per well, from 8 different wells in a row sequence. The first control passage is performed without administering any solution; the second test passage is performed after administering either a mock solution (typically KRH, control experiment) or solutions containing increasing concentrations of a compound (drug testing experiment). When testing an antagonist, this is administered before starting the procedure. The minimum delay between the administration of a drug and the electric pulse in the test passage is ∼1 s. No subjective intervention is required. A CRC (8–9 points dilution series, calculated from 5 sampling areas including several thousand cells) is obtained in less than 10 minutes.

The above results were part of the original method proposal^10^. However, several aspects hampered its use for drug screening purpose: lack of automation of the procedure, operator-dependent analysis, long execution times, cell exposure to many electric pulses with detrimental effects on its integrity, cell exposure to multiple concentrations of the drug with possible desensitization of the receptor etc. Figure 3a shows a block diagram of *ionChannelΩ*, the platform that was developed to exploit this approach and used in the experiments described below.

The typical protocol for the analysis of a candidate drug requires two passages performed in sequence: the first passage (reference) is performed without administering any compound; the second passage (test) is performed after administering either a mock solution (control experiment) or solutions containing increasing concentrations of a compound (drug testing experiment) (Fig. 3b).

Images are processed off line by a software program developed in MATLAB that offers the possibility to employ various filters, parameters and types of analysis^11^. The ΔF/F_R_ values recorded during our experimental protocol in individual pixels, are represented as frequency distribution graph (Supplementary Figure 2) or treated as ordered pairs graphed in a scatter plot (Supplementary Figure 2) where the slope represents the change in resistance (Rd/Rc). Individual values of change in resistance can be plotted as such or displayed as changes in conductance (Gd/Gc) to obtain semi-logarithmic concentration-response curves (CRCs).

The whole procedure to obtain a, single CRC (8–9 points dilution series, each point calculated from 5 sampling areas including several thousand cells) is obtained in approximately 10 minutes.

### Pharmacological validation of *ionChannelΩ*

The *ionChannelΩ* was tested with cell lines expressing different kinds of ligand-gated receptors.

**Figure 4:**
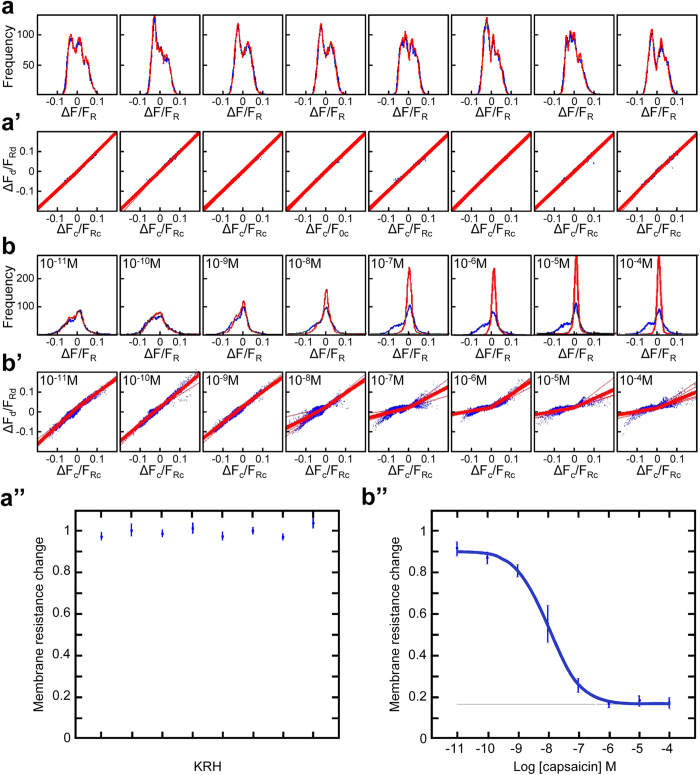
Concentration-response experiments performed with ionChannelΩ. Experiments were performed on CHO cells expressing the hTRPV1 receptor. In each experiment, 5 sampling areas per well (on 8 wells in a row) were studied by *ionChannelΩ*. Frequency distribution, scatter plot, and CRCs are shown for a control experiment (**a**, **a’** and **a”**) and for an experiment in which increasing concentrations of capsaicin (reported for each graph) were administered (**b**, **b’** and **b”**). The blue line in **a** and **b** shows the distribution of ΔF/F_R_ values obtained in the first (reference passage) while the red line shows the distribution recorded in the second (test passage) performed after administering either a mock solution (control experiment, **a**) or solutions containing increasing concentrations of capsaicin (drug testing experiment, **b**). Distributions are superimposed in controls (**a**) but not in the presence of the higher concentrations of capsaicin (molarities are shown in the upper left corner) where ΔF/F_R_ values show a tendency to cluster around zero with the increase in capsaicin concentrations (note the different frequency scale in **a**,**b**). Correlation values for pairs of ΔF/F_R_ measurements (reference and test values recorded within the same individual pixels) are graphed as scatter plots (blue dots; **a’** for the control and **b’** for the capsaicin experiment) where changes in ΔF_c_/F_Rc_ values are expressed in percent. Linear regression lines are drawn for values collected from each sampling area (thin red lines); the thick red line represents the mean of the five lines. Note the different slopes under conditions of depolarization and hyperpolarization for higher concentrations of capsaicin. The slope values of the mean linear regression lines obtained in **a’** and **b’** are employed to obtain the CRCs shown in **a”** and **b”**, respectively (bars represent the standard error of the mean of the five lines values). The concentration-response for capsaicin is represented by a semilogarithmic plot of capsaicin concentrations and fitted with an inverse sigmoid function (**b”**, blue line). These experiments are representative of at least 20 independent experiments for both controls and capsaicin.

The human vanilloid receptor, hTRPV1, is a non-selective cation channel, activated by capsaicin^12^. In full agreement with the theory, in control experiments conducted on a CHO-K1 line expressing the hTRPV1, neither the frequency distribution of ΔF/F_R_ values (Fig. 4a), nor the linear regression of their scatter plot distribution (Fig. 4a’) were changed. In contrast, in the presence of increasing concentrations of capsaicin in the second passage, the frequency distribution was altered stepwise (Fig. 4b) with scatter plots yielding, upon linear regression, a unitary slope resistance progressively reduced up to a final ∼20% of control (Fig. 4b’). Of note, the capsaicin effect appears to be stronger in depolarizing rather than in hyperpolarizing conditions (compare the slope for negative values with that for positive values in Fig. 4b’), an experimental evidence of the well-described outward rectification for this channel^13^.

When the values of resistance changes were plotted as a function of the applied solutions, a flat response was observed in the presence of a mock solution (KRH; Fig. 4a”) whereas a decrease in resistance, described by an inverse sigmoid, was obtained when cells were exposed to a scale of logarithmic concentrations of capsaicin (4b”).

Taking into account that resistance is not linearly related to conductance, values of Rd/Rc can be transformed in Gd/Gc values according to Ohm’s law. Before definitely adopting this approach, we validated it by manual patch clamp to verify whether the extrapolation of R values from the voltage changes elicited by square current pulses, along with their further conversion to G values, was influencing CRCs and thus EC_50_ estimation. Single cells, from the same CHO-K1 line expressing the hTRPV1, were exposed to increasing concentrations of capsaicin and tested, in rapid alternation, in both current and voltage clamp, to mimic our experimental conditions and obtain data in the conventional way, respectively. These combined measurements provided direct evidence that CRCs obtained by the two approaches are superimposable (Supplementary Figure 3) and that values of EC_50_ are not statistically different (687 ± 186 nM in voltage clamp and 774 ± 336 nM in current clamp; n = 5, Supplementary Figure 3).

**Figure 5:**
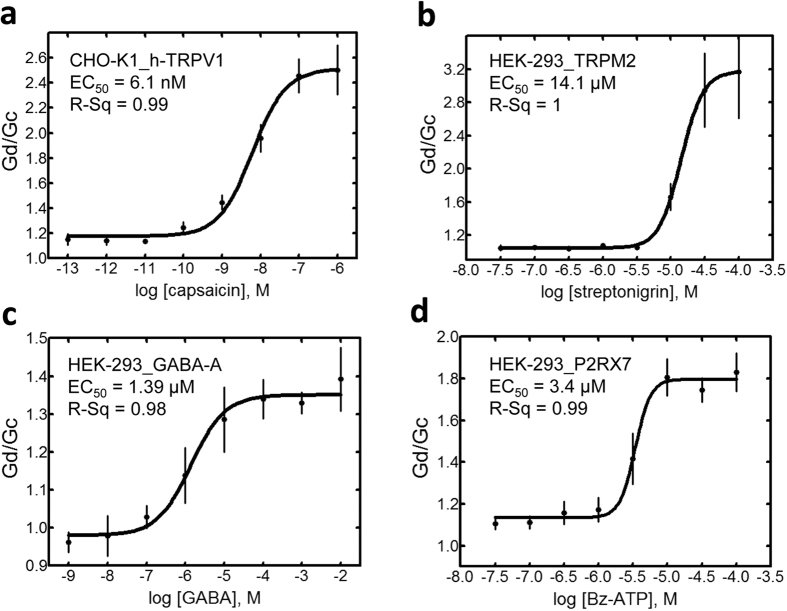
Validation of ionChannelΩ screening system with different ligand-gated ion channels. CRCs are obtained as described in Fig. 4 and represented as change in conductance. The semilogarithmic plots for 4 different drug/receptor interactions are shown: capsaicin/h-TRPV1; streptonigrin/h-TRPM2; GABA/h-GABAA; and Bz-ATP/h-P2 × 7 R. Data obtained as in Fig. 4 are converted, represented by a semilogarithmic plot of drug concentrations and fitted with a sigmoid function. R-Sq, as a measure of the goodness-of-fit, and EC50 are reported in the panels. These experiments are representative of at least 7 independent experiments for each condition.

Having established the validity of the approach, all CRCs were then expressed as changes in conductance. The capsaicin EC_50_ calculated by *ionChannelΩ* was 6.1 nM (Fig. 5a); on the same CHO-K1 line expressing the hTRPV1 an EC_50_ of 11.75 nM was estimated by FLIPR (Screen Quest™ Fluo-8).

The same type of analysis was performed on the transient potential receptor melastatin-2, TRPM2, a non-selective, calcium-permeable cation channel activated by ADP-ribose (ADPR) and modulated by a number of physiological processes^14^. HEK-293 cell expressing the TRPM2 channel were challenged by streptonigrin, a potent agonist for these channels. Figure 5 b shows the CRC with an EC_50 _ = 14.1 μM; when the same cell line was analysed by FLIPR (Calcium Assay Kit), an EC_50 _ = 4.3 μM was obtained. Values obtained by *ionChannelΩ* and FLIPR are comparable and produce potency estimations higher than those obtained by QPatch 16X.

Next, we studied a HEK-293 cell line expressing the GABAA receptor, a ligand-gated ion channel that is selectively permeable to chloride and that can be hardly studied for drug screening purpose by fluorescent based assays. Also in this case, a CRC was obtained by *ionChannelΩ*, with a measured EC_50_ = 1.39 μM for GABA (Fig. 5c). This value compares with the EC_50_ = 4.7 μM obtained by FLIPR (BLUE; Membrane Potential Assay).

Finally, we focused our experiments on P2RX7, an ion channel that shows an initial small non-selective cation conductance^15^, followed by increased membrane permeability to large organic cations upon prolonged and repetitive agonist stimulation^16^. We characterized the activity of BzATP, a potent and specific agonist of the P2RX7 on a HEK-293 cell line expressing the receptor; Fig. 5d shows a CRC obtained by the *ionChannelΩ* with an EC_50_ = 3.4 μM. The parallel analysis by FLIPR (GCaMP2.1 calcium biosensor) gave a figure of 6.35 μM.

### Assay precision and reproducibility of *ionChannelΩ*

**Figure 6:**
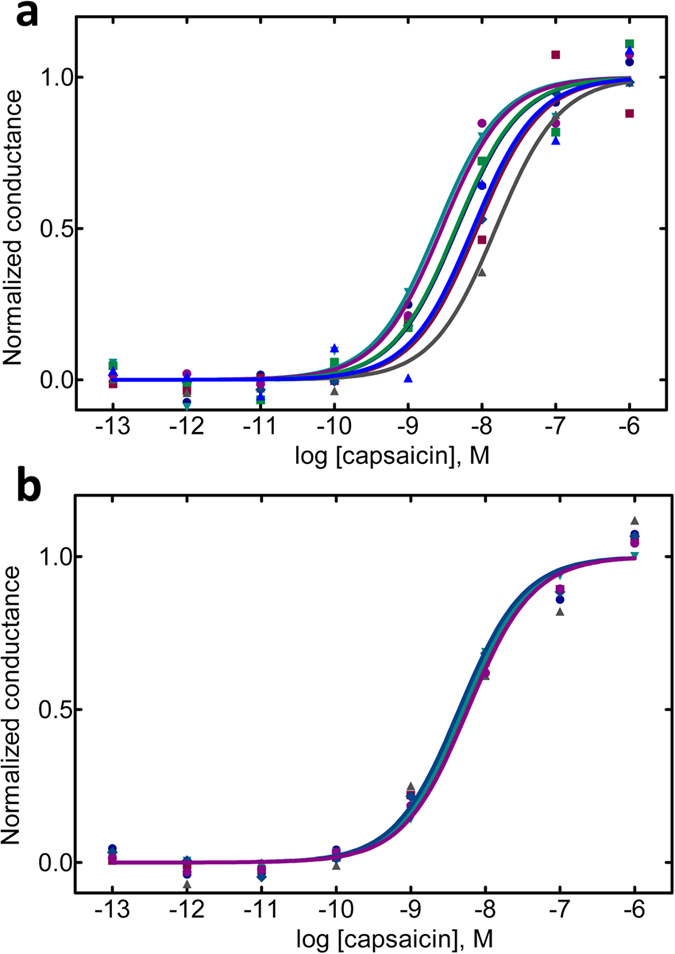
Intra-multiwell and inter-multiwell variability of *ionChannelΩ*. Independent CRCs were obtained for capsaicin on CHO-cells expressing the hTRPV1 in full automation. Panel a shows the 8 curves obtained in sequence from the 8 lanes of a single multiwell. Panel b shows the superimposition of 6 curves (each is the mean of the 8 curves obtained in a single multiwell) obtained from 6 independent multiwells in different days. Curves are represented as conductance changes, fitted with a nonlinear regression algorithm and normalized (Min-Max).

Ten independent multiwells were analysed in 6 experimental days, obtaining a total of 80 CRCs for capsaicin. By computing membrane conductance values obtained at the lowest and the maximal concentrations in all CRCs, a Z factor of 0.42 was estimated. Figure 6 shows 8 CRCs recorded from a single multiwell (a) and 6 CRCs representative of 6 independent multiwells (b; each curve is the mean of the 8 curves obtained in one of the 6 multiwells). Intra-plate (rows-to-rows) and inter-plate (plate-to-plate) consistency of CRCs was further estimated according to the criteria proposed by the National Center for Advancing Translational Sciences of the NIH^17^. The analysis of intra-multiwell variability gave the following results: maximum Coefficient of Variation (CV_max_) = 6.7% (suggested <20%); mean EC_50_ = 6.7 nM with a max variation of 1.24 fold change (suggested <2); Z’ factor: 0.69 between min and max signals (suggested >0.4). The study of inter-multiwell variability was conducted on 8 CRCs from 6 independent MWs investigated in different days. The 6 independent MWs show a mean EC_50_ = 5.16, with a variation of 0.14 fold change (suggested <2).

Finally, *ionChannelΩ* was challenged with a panel of 30 candidate antagonists of the P2RX7 receptor and the results were double blind compared with those obtained by both FLIPR and QPatch 16X (ion current measurements). In these experiments, the antagonists were administered before the second test passage, in which cells were exposed to the agonist BzATP (20 μM).

Table 1 compares the IC_50_ of the 7 molecules that showed some degree of antagonistic activity with at least one of the assays. Overall, IC_50_ values obtained by *ionChannelΩ* and FLIPR are comparable and produce potency estimations somewhat lower than those obtained by QPatch 16X.

## Discussion

An electro-optical approach that combines electrical field stimulation with detection of changes in membrane potential by voltage-sensitive dyes was proposed for the study of voltage-gated channels^9^. This methodology, which shares aspects of current-clamp electrophysiology, shows attractive features when compared to other ion-channel assays, but is restricted to the study of voltage-gated channels. We propose and validate a new, reliable electro-optical approach for the study of ligand-gated channel activity that uses test current pulses to alter the cellular electrochemical equilibrium and that extrapolates values of membrane conductance variations by the measure of the local changes in membrane potential obtained before and after administration of a drug^10^.

Compared with assays based on the fluorescence measurement of ion concentrations, it is of wider applicability, since it is limited neither by the nature of the flowing ion nor by the intensity of the electrochemical gradient at rest. It is also more informative, since it provides a straightforward estimation of ion channel activity and not a measure of downstream events (e.g. calcium-induced calcium release). Our approach has distinct advantages even when compared with simple optical measurements of changes in membrane potential, an indirect, non-linear readout of the channel activity^7^. In fact, the modest efficiency of fast VSD severely limits the signal-over-background detection, particularly when channels pass extremely small currents. On the other hand, slow VSD, more sensitive to changes in membrane potentials, have a low temporal resolution most often incompatible with channel kinetics. Our approach, by increasing the driving force of ions able to flow through the open channels, makes it possible to use fast VSD and to unmask those currents, such as chloride, that are negligible at resting membrane potential and therefore still represent a technical challenge^18^.

We provide a direct comparison of *ionChannelΩ* with both FLIPR assays and electrophysiology, widely considered the gold standard for the study of ion channel activity^3^. Manual patch-clamp has very low throughput and requires highly skilled and trained personnel. The development of automated patch-clamp has helped to mitigate these constraints while offering adequate data consistency and success rate^19^. Moreover, advances in microfluidic design have opened the possibility to study also ligand-gated ion channels^20^. However, also conventional whole-cell patch-clamp has major drawbacks that limit the value of the technique: washout of intracellular constituents, with consequent current rundown, and disruption of intracellular homeostasis, in particular calcium homeostasis (see an exhaustive review of these aspects in ref. 21). Therefore, the subsequent application of increasing doses of a drug can be accompanied by significant current loss as well as alterations in channel gating properties^22^. Our electro-optical approach, inspired by electrophysiology, has the advantage of fully preserving the integrity of the plasma membrane; accordingly, it is expected to provide a more realistic view of the cellular events occurring during ion channel activation. Overall it appears that ionChannelΩ and FLIPR data are comparable and produce potency estimations somewhat different with respect to those obtained by QPatch16X. In some case the potency estimated by QPatch16X appears to be higher in others lower. For instance, we observed for capsaicin EC_50_ values in the 10^−8^ M range, in agreement with data we obtained by FLIPR, as well as others laboratories^23^,^24^,^25^,^26^,^27^. Interestingly, a similar range of EC50 values was also obtained with Ca^45^ 28 and perforated patch^29^. In contrast, our manual and automated patch-clamp data, in agreement with those reported by the literature^13^,^30^,^31^, consistently show an EC50 in the 10^−6^ to 10^−7^ M range. Overall, these observations raise the possibility that alterations of plasma membrane integrity and/or of second messenger signalling cascades, might significantly influence drug potency estimation. A biased estimate of EC50 can also be caused by desensitization, a condition in which responsiveness of the receptors is diminished upon prolonged exposure to their agonist. For instance, h-TRPV1 is known to undergo strong desensitization, which is even faster at low agonist concentration^32^,^33^,^34^. This is not a limitation for the procedure we propose, since exposure to the agonist can be as short as one second and no multiple exposures to the agonist are required to obtain a CRC.

Clearly, our procedure cannot compete with the electrophysiology approaches in terms of temporal resolution of kinetics and voltage control; however, it is significantly faster, does not alter cytosolic composition and does not require specific expertise. Most importantly, the proposed assay shares the capability of providing direct information not only on channel conductance but also on its dependence on membrane potentials. This latter aspect is a biophysical hallmark of several channels that display different ability to sustain inward and outward ionic currents. h-TRPV1 are known to exhibit outward rectification^13^ and this property was confirmed by our scatter plots, where resistance/conductance values are less affected by capsaicin in hyperpolarizing conditions.

In order to be accepted, a new assay must be validated and must prove to be robust. Although the theory behind the assay is rather straightforward, we verified whether changes in potential elicited by a constant current pulse could be transformed in conductance values without significant deviation from direct measurements of current. The consistency of CRCs and EC_50_ values obtained by current clamp and voltage clamp within the same cells fully support our approach. Furthermore, the whole process performed in full automation with no subjective intervention of the operator, was tested by three lines of experiments: (1) qualitative benchmarking on different types of ligand-gated channels; (2) quantitative evaluation of intra-plate and inter-plate variability of the assay; (3) blind comparison with the reference assays QPatch 16X and FLIPR, on a small panel of antagonists designed to modulate P2RX7 receptors activity. The experiments performed on TRPV1, TRPM2, GABAA and P2RX7 receptors clearly show the broad applicability of this procedure and the EC_50_ values obtained are in good agreement with those reported by FLIPR. The reproducibility was further tested by evaluating capsaicin potency on TRPV1, an ionic channel that shows rectification and can be influenced by environmental changes in temperature and pH. The assay demonstrates intra-plate and inter-plate variabilities within the confidence intervals indicated in the NIH guidelines for drug screening^17^. Finally, also the simulation of the use of the platform in a drug screening context provided positive results: the potencies estimated for a small number of new putative antagonists for the P2RX7 were in accordance with those obtained by FLIPR and somewhat lower than those measured by QPatch 16X. In any extent, a good agreement in the rank order of potencies was observed for all the assays.

In conclusion, we demonstrate that this approach can measure changes in membrane conductance and is limited neither by the specific permeability of the ion channels nor by the extent of the ionic fluxes at rest. Accordingly, we propose *ionChannelΩ* as a new automated platform for the high content study of molecules acting on ligand-gated ion channels, in a time- and cost-effective way.

## Methods

### Materials

Krebs-Ringer’s solution buffered with HEPES (KRH) composition (mM): 130 NaCl, 5 KCl, 1.2 MgSO4, 1.2 KH2PO4, 2 CaCl2, 6 glucose and 25 HEPES/Na (Lonza), pH 7.4. di-4-ANEPPS was from Invitrogens. D-PBS w/o Ca^2+/^Mg^2+^ from Euroclone. Standard Tyrode’s Buffer (mM): 130 NaCl, 5 KCl, 2 CaCl2, 5 NaHCO3, 1 MgCl2, 20 HEPES, pH 7.4. Low Calcium, Magnesium free Tyrode’s Buffer, 130 NaCl, 5 KCl, 0.3 CaCl2, 5 NaHCO3, 20 HEPES, pH 7.4. The following drugs are from Sigma: Streptolysin O, γ-Aminobutyric acid (GABA), streptonigrin and 2′(3′)-O-(4-Benzoylbenzoyl)adenosine 5′-triphosphate triethylammonium salt (Bz-ATP). The panel of candidate antagonists of the P2RX7 ion channel were from Centro Interdipartimentale Studi bio-molecolari e applicazioni Industriali - CISI (Milan, Italy).

All drugs and di-4-ANEPPS were dissolved in DMSO and stored at −20 °C until use. Drugs were diluted in KRH to give the proper concentrations; cells were exposed to a maximal DMSO concentration of 0.5%.

Media and supplements to grow cells: DMEM and DMEM-F12 1:1 Mix with Ultraglutamine I (Dulbecco’s Modified Eagle’s Medium and Nutrient Mixture F-12; Lonza); EMEM (Eagle’s Minimum Essential Medium) with Earle’s Balanced Salt Solution (Lonza); EX-CELL ACF CHO medium (Sigma). Fetal Bovine Serum (FBS) (Euroclone), Sodium Pyruvate Solution (Lonza); Sodium Bicarbonate Solution (Lonza); Ultraglutamine I (Lonza); L-Glutamine (GIBCO) Penicillin/Streptomycin Solution (Lonza), G418 Disulfate Salt Solution (Sigma), Poly-L-Lysine Hydrobromide (Sigma). Trypsin-EDTA and Soybean trypsin inhibitor were from Sigma.

### Cell cultures

Cell lines were grown at 37 °C in a 5% CO_2_ humidified atmosphere. Axxam provided the following lines: Chinese Hamster Ovary cells expressing the human vanilloid receptor-1 (CHO-K1_TRPV1), HEK-293 expressing the human TRPM2-R (HEK-293_TRPM2), the human GABAA-R (HEK-293_GABA-A) and the human P2 × 7-R (HEK-293_P2RX7).

HeLa cells were grown in DMEM supplemented with 10%FBS, 1% L-glutamine and 100 units/ml penicillin/streptomycin. CHO-K1_TRPV1 were cultured in DMEM-F12 1:1 Mix with Ultraglutamine I supplemented with 1 mM sodium pyruvate solution, 13 mM HEPES Buffer, 0.375% sodium bicarbonate solution, 10% FBS, 1% penicillin/streptomycin solution, 1 mg/mL G418. All HEK-293 cells were cultured in EMEM supplemented with 2 mM ultraglutamine1, 10% FBS, 1% penicillin/streptomycin, 200 μg/ml G418.

Two days before the experiments, cells were plated on 25 mm diameter glass coverslips or plastic bottom 96-well black microplates (M0562, Greiner CELLSTAR^®^ 96, Sigma-Aldrich). HEK-293 cells were plated on 96-well microplates coated with 100 μg/ml Poly-L-Lysine.

### Electrical stimulation

Test current pulses (mono or biphasic) were administered to cells by parallel electrodes placed on the plane of the cells. Pulse length was several times the membrane time constant of the investigated cells in order to acquire images at a time of the pulse when plateau was fully established. Current intensity was set high enough to generate significant variations in the fluorescence signal, still avoiding membrane electroporation^35^. More specifically, a plot of the ΔF_c_/F_Rc_ values obtained at increasing current values was obtained: only current intensities giving correlative values lying on the linear part of the plot were used.

### Conventional Microscope

#### Hardware

Cells plated on 25 mm diameter glass coverslips were mounted in a chamber equipped with a pair of platinum wires and loaded with di-4-ANEPPS. The cell chamber was transferred into an inverted microscope (Axiovert 200, Zeiss) equipped with a “Plan-Apochromat” 40x, 1.3 N.A. oil-immersion objective lens (Zeiss), a dichroic filter (565 DCLP, Chroma), a band-pass emission filter (650/13 nm BrightLine Semrock) and a 532 nm laser (30 mW; GLML4-SERIES, Roithner LaserTechnik). Images were acquired by a CMOS camera with integrated intensifier (Fastcam 512 PCI, Photron). The stimulation unit (SIV-102, Warner Instrument) was synchronized with image acquisition by a synchronization unit (STG2004, Multi Channel System).

#### Image analysis

Images were processed off-line by an image analysis program developed in MATLAB (The MathWorks). In brief: after correction of possible shifts, a functional mask was generated to select pixels with evoked membrane potential changes higher than a predefined threshold. Based on this mask, the operator subjectively selected regions of interest (ROIs) highly responsive to electric stimulation. Fluorescence intensity values were calculated as the mean of the values obtained in the selected ROIs within the 3 stimuli series administered in the different conditions (before and during the plateau phase of the pulse, in the absence and in the presence of a test compound). The CRCs were drawn by interpolating changes in ΔF/Fr values as described.

### IonChannelΩ screening system

#### Hardware

Cells plated on multiwells were loaded with di-4-ANEPPS and transferred into ionChannelΩ. The optical system, designed on a classical epifluorescence scheme, is equipped with a 532 nm laser (CRL-CL532-050-L, CrystaLaser), an objective lens (Plan-Apochromat 20x/0.8, Zeiss) moved by a piezoelectric actuator (Pifoc P-725 Physik Instrumente), a dicroic mirror (T560LPXR, Chroma Technology Corporation) and a band-pass filter (BrightLine HC 661/20, AHF). A motorized stage provides flexible and accurate positioning of the multiwell plates (H101A, Prior Scientific). A linear actuator (M-683.2U4 Physik Instrumente) controls the positioning of the electrodes that convey the constant current pulses generator (2602 A, Keithley SourceMeter, Tektronix Company). Finally, a carthesian liquid handler dispenses the solutions (TriTon Liquid Handling (XYZ) Robot, Tricontinent Scientific). Images were acquired by an EM-CCD camera (512 × 512, 16-bit mono- chrome, C9100-13 ImagEM Hamamatsu Photonics).

#### Software

The control system is based on a modular architecture composed of a subset of autonomous routines that control specific tasks of the platform. A graphic interface allows the operator to create and execute a sequence of operations with full control on specific variables (e.g. acquisition time; pulse shape, length and duration; delays, etc).

#### Screening performance

The reproducibility of focus is better than 500 nm; XY repositioning between passages is governed by hardware and controlled via software (cross-correlation of images) with repositioning error below pixel size.

The screening, performed in full automation, includes: auto focus, electric pulse delivery, drug administration, image acquisition/analysis and calculation of CRCs.

#### Image analysis

Images are processed off-line by a MATLAB-based Image Analysis program, developed in the laboratory, which offers the possibility to employ different filters, parameters and types of analysis in order to extract the data necessary to draw CRCs. By using a Design of Experiment approach, a simple and robust configuration was adopted, mainly based on image binning (4X to reduce noise) and a shape-mask^11^. More specifically, pixels were selected as part of the shape mask when the following requirements were met: to be part of the cell (recognition of object boundary from fluorescence signals); to be confined to the plasma membrane region (calculation of a ring of depth boundaries). The ΔF/F_R_ values were calculated within all of the individual pixels that constitute the shape mask and were represented as frequency distribution graph or treated as ordered pairs graphed in a scatter plot where the slope represents the change in resistance (Rd/Rc). Individual values of change in resistance were plotted as such or transformed in changes in conductance (Gd/Gc). In either case, mean values ± standard error (calculated from values obtained in the different sampling areas of the same well) were obtained at the various concentration of the drug and fitted by a nonlinear regression curve. The CRCs show on the X axis the logarithmic concentration of the drug and on the Y axis either the change in resistance or in conductance.

Statistical tests were carried out on GraphPad Prism 5.01 (GraphPad Software); the Z-factor was calculated according to Zhang^36^.

### FLIPR

Intracellular calcium concentration was measured by: GCaMP2.1 calcium biosensor for P2RX7; Screen Quest™ Fluo-8 No. Wash Calcium Assay Kit (AAT Bioquest) for TRPV1; and Calcium Assay Kit (Molecular Devices) for TRPM2.

Membrane potential was measured for GABA-A assay using the Membrane Potential Assay Kit (BLUE; MP, Molecular Devices).

Loading of the fluorescent dyes and analysis at FLIPRTetra (Molecular Devices) were performed according to manufacturer protocols.

For the P2RX7 assay, cells were preincubated in Low Calcium, Magnesium free Tyrode’s Buffer.

Reference blockers were injected at FLIPRTetra instrument and fluorescence signal was recorded. After 10 minutes a second injection of reference activators was performed and fluorescence was measured.

### Automated patch clamp

#### Instrumentation

QPatch 16X automated electrophysiology platform (Sophion, Biolin Scientific). is a silicon chip-based planar patch clamp device allowing for up to 16 parallel recordings during one experimental run. Cells are added to each well on the QPlate chip and drawn by suction onto a small apertures to obtain a high seal quality between the cell membrane and treated silicon surface, after which a whole-cell recording is achieved by applying a combination of suction and voltage pulses to break through the cell membrane and allow perfusion of the cell interior.

#### Cell preparation

Before the experiments, cells were washed with D-PBS w/o Ca^2+^/Mg^2+^ and detached from the flask with trypsin-EDTA (diluted 1:10). Cells were finally washed with: 25 mL EX-CELL ACF CHO medium; 0.625 mL HEPES; 0.25 mL of 100x Penicillin/Streptomycin; 0.1 mL of Soybean trypsin inhibitor (10 mg/ml). The intracellular solution contained (mM): 135 CsF, 10 NaCl, 1 EGTA, 10 HEPES (pH 7.2 with CsOH). The extracellular solution contained (mM): 145 NaCl, 4 KCl, 0.5 MgCl2, 1 CaCl2, 10 HEPES, 10 Glucose (pH 7.4 with NaOH).

#### Voltage clamp experiments

Standard whole-cell voltage clamp experiments were performed at room temperature using multihole plates (10 holes for each of the 16 wells). Following the establishment of the whole-cell configuration, cells were held at −80 mV and the P2 × 7 current was evoked by applying BzATP (100 μM; for 3 seconds), in the absence (control) or presence of increasing concentrations of the compound under evaluation (3 minutes period pre-incubation). Data were sampled at 2 KHz.

#### Data analysis

For data acquisition and analysis we used the Sophion proprietary and excel software. The percentage of inhibition elicited by each concentration of the compound under investigation was calculated as: %inhibition = 100 − 100 × (IP2 × 7CP/IP2 × 7CT); where IP2 × 7CT and IP2 × 7CP are the inward current elicited by the BzATP in the presence of the vehicle (0.1% DMSO) or after 3 minutes preincubation with increasing concentrations of the compound under investigation, respectively. When the percentage of inhibition elicited by the highest concentration tested was superior to 50%, an IC50 was determined with GraphPad Prism 6.

### Manual patch clamp

#### Instrumentation

HEKA EPC10 digitally controlled amplifier in combination with PATCHMASTER software (HEKA Electronics).

#### Cell preparation

16–24 hours before experiments, cells were seeded onto poly-D-lysine coated coverslips (Warner instruments, VWR international). Before experiments, cells were washed with extracellular solution and put into the recording chamber.

#### Patch clamp protocols

Standard whole-cell voltage clamp experiments were performed at room temperature. Capacitative currents were automatically subtracted, data were filtered at 2.9 KHz (−3dB, 4-pole Bessel lowpass) and digitized at 50 and 100 μs per point, voltage-clamp and current clamp, respectively.

#### Data analysis

The raw conductance in V-Clamp (GVC) was obtained as follows:

I(+40mV)/ΔVwhere I(+40 mV) is the current amplitude measured at +40 mV, upon application of the protocol 1, and ΔV is (+40 - Erev.) (reversal potential of the whole-cell currents).

In C-Clamp mode the absolute Vm value was averaged in the last 30 ms of the injection step and then subtracted from the basal Vm measured at the beginning of the protocol. Membrane conductance was calculated as the ratio ΔVm/ΔVm in the presence of increasing concentrations of capsaicin.

Analysis were performed by HEKA proprietary software, Microsoft Excel and fitting of the dose response curves were done by GraphPad Prism.

## Additional Information

**How to cite this article:** Menegon, A. *et al*. A new electro-optical approach for conductance measurement: an assay for the study of drugs acting on ligand-gated ion channels. *Sci. Rep.* **7**, 44843; doi: 10.1038/srep44843 (2017).

**Publisher's note:** Springer Nature remains neutral with regard to jurisdictional claims in published maps and institutional affiliations.

## Supplementary Material

Supplementary Figures 1-3

## Figures and Tables

**Table 1:** IC_50_ values of molecules showing antagonistic activity on P2RX7 receptor.

Compound	ionChannelΩ IC_50_ (μM)	FLIPR IC_50_ (μM)	QPatch 16X IC_50_ (μM)
AXX00179375	8.6	1.59	1.95
AXX00179433	0.31	0.35	0.02
AXX00179853	29.76	27	Inactive
AXX00179861	10.04	1.91	6.51
AXX00179873	3.33	2.2	0.85
AXX00179924	1.77	1.86	1.10
AXX00418759	0.22	0.44	0.02

Within a panel of 30 compounds, designed to act as antagonist on P2RX7 receptor, only 7 showed a measurable activity by one of the following approaches: *ionChannelΩ*, FLIPR, QPatch 16X. The table reports the IC_50_ values (double blind measurements) obtained for these 7 compounds on a cell line expressing P2RX7 receptors. IC_50_ values were calculated by exposing cells to concentrations comprised between 10^−4^ and 10^−9^ M and then challenging them with the agonist BzATP (20 μM). n ≥ 3 CRC.
