# Supplementary material for: A new electro-optical approach for conductance measurement: an assay for the study of drugs acting on ligand-gated ion channels
Source: Sci Rep. 2017 Mar 21;7:44843. doi: 10.1038/srep44843 (PMC5359596; doi:10.1038/srep44843)
Supplement: Supplementary Figures 1-3 [file srep44843-s1.pdf]

A new electro-optical approach for conductance measurement: an assay for the study of drugs acting on ligand-gated ion channels

Menegon, Pitassi, Mazzocchi, Redaelli, Rizzetto, Rolland, Poli, Imberti, Lanati, Grohovaz

Supplementary-figure 1

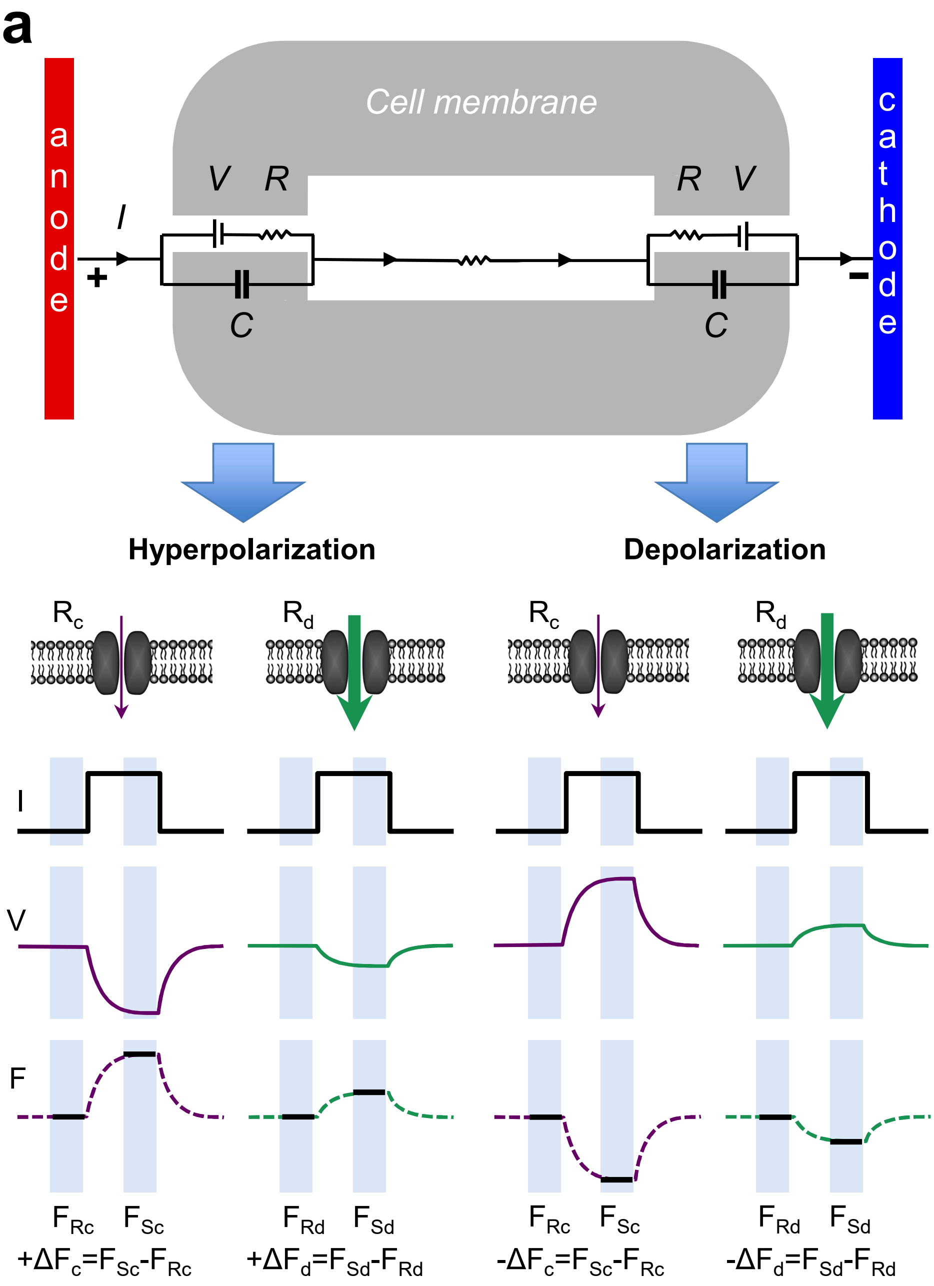

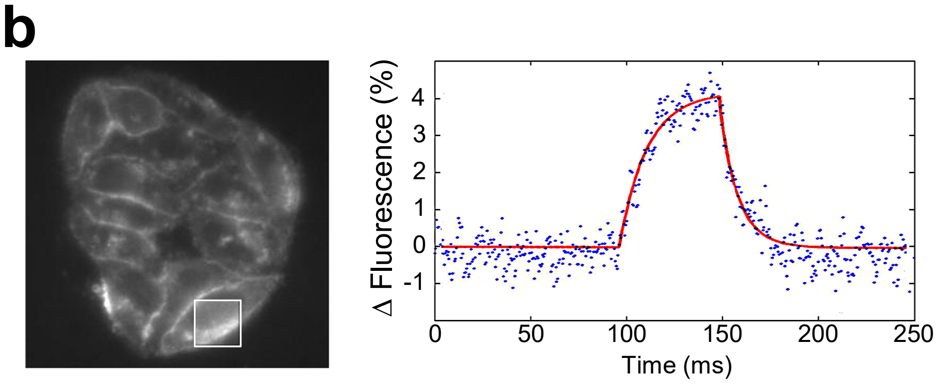

Supplementary Figure 1 - Effects of electric field stimulation on cell membrane potential

The upper panel in **a** shows the equivalent circuit model of a plasma membrane with the pathways of the currents flowing through a cell exposed to an electric field. A square current pulse (I), generated by parallel plate electrodes, induces capacitive currents that hyperpolarize one side of the membrane and depolarize the opposite one. As membrane potential becomes more negative or positive, ionic currents are driven to re-establish the electrochemical equilibrium. The overall consequence of this process is that the change in membrane potential, during the electrical stimulation, reaches a new stable value that depends on the membrane resistance (see V simulated traces). In fact, if the membrane resistance (control resistance, R_c) is reduced by effect of a drug (R_d), the membrane potential variation is more effectively counteracted by ionic currents and a lower plateau is observed for both hyperpolarization and depolarization (compare green with purple V traces). These changes in membrane potential can be monitored by di-4-ANEPPS, a fast VSD that increases its fluorescence upon hyperpolarization and decreases it in the presence of depolarization (see the F simulated traces). The images of fluorescence are acquired at two specific times, highlighted by the light blue intervals (F_R): at rest (F_{RC} or F_{RD}, i.e. in the absence, or presence of a drug, respectively) and during the plateau phase of the stimulus (F_{SC} or F_{SD}). Accordingly, changes in fluorescence can be measured as the difference (ΔF) between these couple of values: ΔF_c or ΔF_d, whether in the absence or in the presence of a drug affecting resistance.

Panel **b** demonstrates the capability of di-4-ANEPPS, a fast VSD, to follow the kinetics of membrane potential changes imposed by a square current pulse in CHO cells. Fluorescence intensity values (dots on the right graph) were collected at high rate (2000 frame/s) and measured within the square highlighted in the image on the left. The response of the membrane potential to the square pulse was as expected from a typical resistor-capacitor (RC) circuit (red line).

Supplementary-figure 2

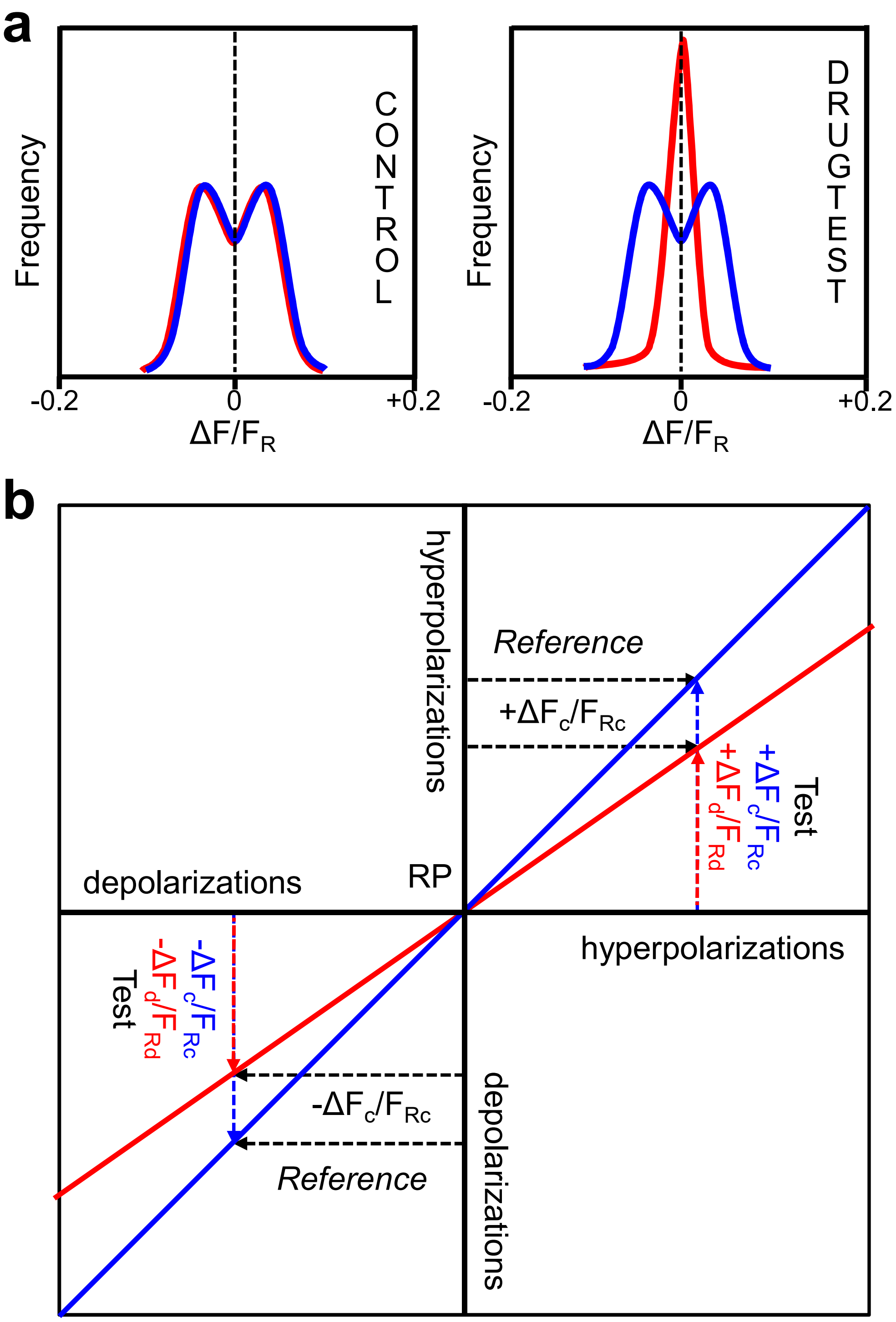

Supplementary Figure 2– Graphic representation of changes in membrane potentials.

The simulations in **a** and **b** show $\Delta F/F_R$ (see Supplementary Figure 1) values represented as either frequency distribution graph (**a**) or ordered pairs graphed in a scatter plot (**b**).

Panel **a** shows the simulation of the frequency distributions of positive and negative $\Delta F/F_R$ values, before (blue line) and after (red line) administration of a test solution (KRH, in "control experiments" and a drug able to determine an increase in membrane permeability, in "drug testing experiments" (left and right panels respectively).

In controls, the $\Delta F/F_R$ values recorded in the reference passage are reproduced also in the test passage with a frequency distribution that is given by the superposition of two bell-shaped patterns crossing to zero (**a**, left panel). If a drug acting on an ion channel is added at the test passage, both positive and negative $\Delta F/F_R$ values are reduced, giving rise to a single narrow peak around zero (**a**, right panel).

In a scatter plot, the correlation values for pairs of $\Delta F/F_R$ (obtained from individual pixels, first at the reference and then at the test passage) are, ideally, laying on a line, whose slope provides a measure of the change in membrane resistance (**b**). In control experiments, reference and test $\Delta F_c/F_{Rc}$ values (blue dashed arrow lines) are, ideally, identical and the slope value is 1 (blue line). In a drug testing experiment, in which membrane resistance is changed, the $\Delta F_d/F_{Rd}$ test values are reduced with respect to the $\Delta F_c/F_{Rc}$ reference values (red dashed arrow line); as a consequence, the lower is the resistance, the closer to zero is the slope value (red line). Changes in $\Delta F_c/F_{Rc}$ values are expressed in percent. Note that when channels have a ohmic behaviour, slope values are the same in both depolarization and hyperpolarization.

Supplementary-figure 3

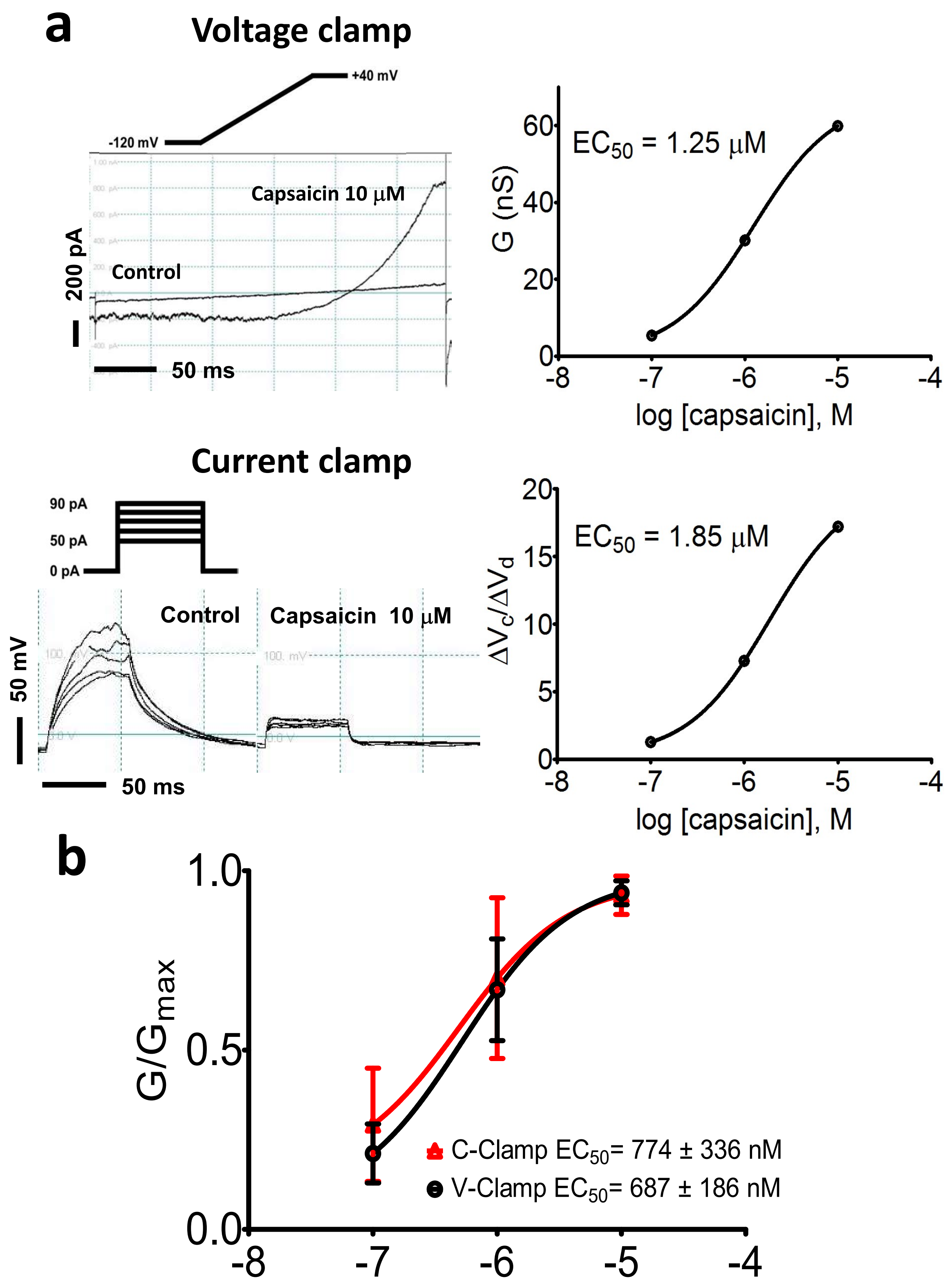

Supplementary Figure 3– Representative examples of current clamp and voltage clamp recordings performed in the same cell

Two consecutive protocols in voltage clamp (upper strip) and current clamp results (lower strips) were applied to the same CHO_TRPV1 cell (**a**).

Protocol 1 (voltage clamp): after achievement of the whole-cell configuration, cell was kept in voltage-clamp mode at -80 mV; then, after a brief (50 ms) pulse at -120 mV a 500 ms-long voltage ramp from -120 to + 40 mV was applied (upper left panel). Finally, cell was kept at +40 mV for 50 ms after the ramp. The entire protocol was applied every second until a stable steady-state current could be recorded. The lower left panel shows representative traces recorded in control conditions and after application of capsaicin (10 μM); a CRC (right panel) was obtained by plotting conductance values (in nS) against the respective concentrations of capsaicin.

Protocol 2 (current clamp): After a stable steady state current was measured in voltage clamp, cells were switched in current clamp mode (I=0). A 50 pA step of current was injected for 100 ms (upper left panel, then the cell was allowed to recover at I=0 for 600 ms. This protocol was repeated five times with incremental current injections of 10 pA (final current injection of +/-100 pA) at 1 Hz. The lower left panel shows representative traces recorded in control conditions and after application of capsaicin (10 μM); after analysis of the traces (see Methods), a CRC (right panel) is shown as the ratio between the conductance (G) normalized to the maximal conductance (Gmax).

The two protocols were repeated in the presence of three increasing concentrations of capsaicin (0.1, 1 and 10 μM).

Panel **b** shows CRCs of capsaicin recorded in voltage clamp and current clamp (black and red, respectively). The responses are both shown as G/Gmax. Each point represents the average ± SEM (n=5).

Analysis: The raw conductance in voltage clamp was obtained by the relation $I_{(+40mV)} / \Delta V$ where $I_{(+40mV)}$ is the current amplitude measured at +40 mV, upon application of the protocol 1, and ΔV is (+40 - $E_{rev.}$) (reversal potential of the whole-cell currents).

In current clamp mode, the absolute Vm value was averaged in the last 30 ms of the injection step and then subtracted from the basal Vm measured at the beginning of the protocol. Membrane conductance was calculated as the ratio between ΔVm in vehicle and ΔVm in the presence of increasing concentrations of capsaicin.
